# Serum Catestatin Levels Correlate with Ambulatory Blood Pressure and Indices of Arterial Stiffness in Patients with Primary Hypertension

**DOI:** 10.3390/biom12091204

**Published:** 2022-08-30

**Authors:** Marko Kumric, Josip Vrdoljak, Goran Dujic, Daniela Supe-Domic, Tina Ticinovic Kurir, Zeljko Dujic, Josko Bozic

**Affiliations:** 1 Department of Pathophysiology, University of Split School of Medicine, 21000 Split, Croatia; 2 Clinical Department of Diagnostic and Interventional Radiology, University Hospital of Split, 21000 Split, Croatia; 3 Department of Health Studies, University of Split, 21000 Split, Croatia; 4 Department of Medical Laboratory Diagnostics, University Hospital of Split, 21000 Split, Croatia; 5 Department of Endocrinology, Diabetes and Metabolic Diseases, University Hospital of Split, 21000 Split, Croatia; 6 Department of Integrative Physiology, University of Split School of Medicine, 21000 Split, Croatia

**Keywords:** hypertension, catestatin, chromogranin A, cardiovascular, biomarker, catecholamines

## Abstract

Accumulating data suggests that catestatin, an eclectic neuroendocrine peptide, is involved in the pathophysiology of primary hypertension (PH). Nevertheless, clinical studies concerning its role in PH are still scarce. Therefore, in the present study, we aimed to explore an association between serum catestatin levels, ambulatory blood pressure (BP) and arterial stiffness in patients with PH and healthy controls. In this single-center study, 72 patients aged 40–70 diagnosed with PH, and 72 healthy controls were included. In patients with PH, serum catestatin concentrations were significantly higher in comparison to the healthy controls (29.70 (19.33–49.48) ng/mL vs. 5.83 (4.21–8.29) ng/mL, *p* < 0.001). Untreated patients had significantly higher serum catestatin than patients treated with antihypertensive drugs (41.61 (22.85–63.83) ng/mL vs. 24.77 (16.41–40.21) ng/mL, *p* = 0.005). Multiple linear regression analysis showed that serum catestatin levels retained a significant association with mean arterial pressure (β ± standard error, 0.8123 ± 0.3037, *p* < 0.009) after model adjustments for age, sex and body mass index. Finally, catestatin levels positively correlated with pulse wave velocity (r = 0.496, *p* < 0.001) and central augmentation index (r = 0.441, *p* < 0.001), but not with peripheral resistance. In summary, increased serum catestatin concentration in PH, predominantly in the untreated subgroup, and its association with ambulatory BP and arterial stiffness address the role of this peptide in PH.

## 1. Introduction

Affecting more than a quarter of the world’s population and representing the biggest single risk factor contributing to the global all-cause mortality, hypertension represents a major healthcare issue [1,2]. The pathophysiological mechanisms underlying primary hypertension (PH) are complex and act on a genetic background [3]. However, despite numerous studies aimed at deciphering the relative contribution of different mechanisms to the development of hypertension, the failure of interventional studies in applying these findings shows that we are still far from understanding the true nature of PH [4]. In recent years, the role of sympathetic nervous system (SNS) dysfunction in PH has gained particular attention, especially with regard to renal sympathetic activity, given that this mechanism has been therapeutically challenged [5]. Yet, although initial trials yielded promising results, the SIMPLICITY HTN-3 trial did not demonstrate the benefit of renal artery denervation for reducing ambulatory blood pressure (BP) [6]. Nevertheless, the failure of this technique in BP reduction does not undermine the importance of SNS activity in PH, it merely demonstrates that its etiopathogenesis requires further insight.

The correlation between arterial stiffness and hypertension, especially in relation to the age-related progression of arterial stiffness, has been well established [7]. Moreover, recent population-level data suggests that arterial stiffness is an independent risk factor for cardiovascular disease and hypertension [8,9,10]. The mechanisms that underlie this association are diverse and include uric acid, aging, vascular calcification and many others [11]. 

Catestatin, a neuroendocrine peptide derived from Chromogranin A, was recently assumed to have a role in the adjustment of BP and, therefore, in the pathophysiology of arterial hypertension as well [12]. Namely, the primary role of this peptide is the negative regulation of catecholamine spillover via neuronal nicotinic cholinergic receptor (nAChR) antagonism [13]. Considering the importance of SNS activity in PH pathophysiology, several authors have explored the role of catestatin in this setting [14,15,16,17]. However, despite the involvement of catestatin in arterial hypertension pathogenesis being undisputed, clinical studies concerning catestatin in PH are still scarce.

Hence, in the present study, we aimed to explore an association between serum catestatin levels and ambulatory BP levels in patients with hypertension and healthy controls. Furthermore, differences in serum catestatin concentrations between treated and untreated patients with PH were explored. Finally, additional aims were to explore an association between serum catestatin levels and indices of arterial stiffness and cardiovascular risk.

## 2. Materials and Methods

### 2.1. Study Design and Ethical Considerations

The present study was designed as a single-center cross-sectional study. The complete course of the study was performed at the Department of Pathophysiology and the Department of Integrative Physiology at the University of Split, School of Medicine (Split, Croatia) from February 2022 to July 2022.

The study was approved by the Ethics Committee of the University of Split, School of Medicine and was conducted in accordance with all ethical principles of the Helsinki Declaration of 2013. Prior to study enrolment, each participant was informed about the procedures, course, aim and potential complications of this research, and each personally signed an informed written consent form.

### 2.2. Subjects and Inclusion/Exclusion Criteria

In the present study, we consecutively enrolled 72 patients aged 40–70 diagnosed with PH and 72 healthy controls. All patients included in the study were diagnosed according to the European Society of Cardiology (ESC) guidelines for management of arterial hypertension [18]. Of the included 72 patients, 38 patients were untreated, whereas 34 patients were treated using angiotensin-converting enzyme (ACE) inhibitors with or without thiazide diuretics or a combination of ACE inhibitors with calcium channel blockers with or without thiazide diuretics.

The inclusion criteria were: Mild (Grade 1) hypertension (140/90 to 159/99 mmHg) or moderate (Grade 2) hypertension (160/100 to 179/109 mmHg); body mass index (BMI) between 18.5 and 30 kg/m^2^; age between 40 and 70 years; and undergoing < 150 min of moderate-to-vigorous activity per week.

Exclusion criteria were: severe (Grade 3) hypertension (>180/110 mmHg); secondary hypertension (including obstructive sleep apnea); hypertensive crisis in the past year; positive smoking status and/or use of vapor-based products and/or a medical or recreational cannabis user; active malignant disease; gout; chronic kidney disease; chronic gastrointestinal disease (e.g., inflammatory bowel disease); current diagnosis or history of any seizure disorder; heart failure; cirrhosis; diabetes mellitus; pregnancy or breast feeding; history of opioid use; dual blood pressure therapy other than ACE inhibitors with diuretic or ACE inhibitors with calcium channel blockers (CCBs) with or without diuretics (e.g., ACE inhibitors with beta blockers); use of any other medications; and unwilling or unable to execute the informed consent documentation. The above noted inclusion and exclusion criteria were assessed with a medical screening questionnaire during a clinical examination and related baseline blood chemistry results.

### 2.3. Clinical and Laboratory Evaluations

Physical examination and relevant items from past medical history were collected from all patients during the screening visit. An altitude meter (Seca, Birmingham, UK) was used to measure height, and body mass was measured using the bioelectrical impedance scale Tanita DC-360 S (Tanita, Tokyo, Japan). BMI was calculated by dividing the value of body mass (kg) and the squared value of height (m^2^). The waist circumference was measured at the level of the midline between the bottom line of the rib arch, in the mid-axillary line, and the tip of the iliac crests. The hip circumference was measured at the level of the largest circumference above the line joining the great trochanters of the femur. Patients were standing upright during both measurements. The waist-to-hip ratio was calculated by dividing measured waist and hip circumference. Office BP during the screening visit was measured according to guidelines for BP measurement, using WatchBP Home A (Microlife AG Swiss Corporation, Widnau, Switzerland) [18].

From 117 volunteers with hypertension that were screened, 72 patients were eligible for study enrollment. Furthermore, the control group was established during a screening visit identical to that of hypertensive patients. Subsequently, all included participants were invited to the premises of the Department of Pathophysiology for the experimental visit. Participants were instructed to report to the Department after an overnight fast (>10 h) and around 07:30. At the start of the experimental visit, office BP was measured in the same manner as during the screening visit. Subsequently, a sample of venous blood was obtained from the cubital vein using a sterile disposable needle. A maximum of 22 mL of blood was drawn, and the whole process of blood sampling was performed by a trained laboratory technician. Part of the sampled blood was immediately analyzed, whereas part of the sample was aliquoted and stored at −80 °C for subsequent analysis of biomarkers, including catestatin. 

Serum catestatin (Phoenix Pharmaceuticals Inc., Burlingame, CA, USA) concentrations were determined by an enzyme-linked immunosorbent assay (ELISA). The reported sensitivity of the assay kit for catestatin was 0.05 ng/mL, with a linear range of 0.05–0.92 ng/mL, and a cross-reactivity with endogenous human catestatin of 100% (intra-assay and inter-assay coefficients of variability were <10% and <15%, respectively). All blood samples were analyzed in the same certified institutional biochemical laboratory, using standard operating procedures. The biochemist was blinded to the participant’s assignment to the experimental or control group.

Finally, participants were instrumented with the 24 h ambulatory BP monitor system Schiller BR-102 plus PWA (Schiller AG, Baar, Switzerland), which was used to assess continuous blood pressure measurements away from the laboratory. The device was set to take measurements every 30 min through the day (8 am–11 pm) and every hour through the night (11 pm–8 am) [10]. Participants were asked to keep their arm still and at the level of their heart when the device started to take a reading. In addition, all patients were required to fulfill a diary of physical activity during the day of the ambulatory BP measurements. Apart from ambulatory BP values, output from the BP monitor systems was pulse wave analysis (PWA). Specifically, pulse wave velocity (PWV), central augmentation index at 75 beats-per-minute (cAIx-75) and peripheral resistance (pRes) were calculated.

### 2.4. Sample Size Calculation

The sample size analysis was conducted before the study onset, using data from a pilot study on 10 healthy subjects and 10 randomly selected subjects from the patient population. The value of serum catestatin levels, which was the main outcome of the study, was used for the calculation. A sample size of 34 participants per group was indicated as significant to detect a standardized difference with 90% power and a type I error of 0.05.

### 2.5. Statistical Analysis

MedCalc Statistical Software version 20.113 (MedCalc Software BV, Ostend, Belgium) and SigmaPlot version 14 (Systat Software Inc., San Jose, CA, USA) were used for statistical data analysis and graph design. Quantitative data was expressed as mean ± standard deviation (SD) or median and interquartile range (IQR), as appropriate, whereas qualitative data was expressed as a whole number and percentage. The Shapiro–Wilk test was used to estimate the normality of data distribution. For the comparison of qualitative variables, the Chi-squared (χ^2^) test was used. For the comparison of quantitative variables, the Student’s t-test for independent samples and Mann–Whitney U test were employed, as appropriate. In addition, to establish whether the observed difference in serum catestatin concentrations between treated and untreated group was independent, we employed non-parametric ANCOVA (Quade method). To assess the relationship between different parametric and non-parametric variables, a Spearman rank correlation analysis was employed. To assess the association between systolic BP (SBP) and catestatin levels, patients were divided into three distinct groups. The groups were based on terciles of catestatin concentrations, and SBP values were subsequently compared with one-way ANOVA with a *post hoc* Tukey test. Finally, multiple linear regression analysis was used to determine the relative importance of independent variables (age and BMI) in the prediction of catestatin serum concentrations. For the detection of multicollinearity in the linear regression analysis, the variance inflation factor (VIF) was used. Statistical significance was set at *p* < 0.05 for all comparisons.

## 3. Results

The comparison of basic characteristics including age, sex, anthropometric data, past medical history, medications and relevant laboratory findings between patients with PH and healthy controls are presented in Table 1. Notably, the hypertension group had significantly higher SBP levels (*p* < 0.001), diastolic BP (DBP) levels (*p* < 0.001), mean arterial pressure (MAP) (*p* < 0.001), PWV (*p* < 0.001), total cholesterol (*p* = 0.045) and triglycerides (*p* = 0.014). There were no significant differences with respect to other presented characteristics.

The comparison of ambulatory BP values between the treated and untreated group of patients with hypertension is presented in Table 2. It was established that the untreated group of patients had a significantly shorter time since the diagnosis of hypertension (*p* < 0.001). There was no significant difference with respect to the other presented variables.

**Figure 1:**
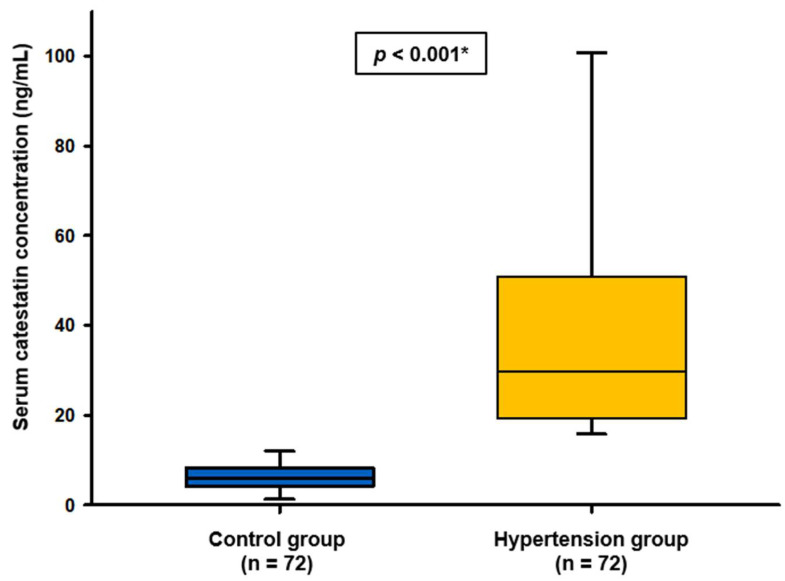
Comparison of catestatin concentrations between patients with primary hypertension and healthy controls. Data are presented as median and interquartile range. * Mann–Whitney U test.

In patients with PH, serum catestatin concentrations were significantly higher in comparison to healthy controls (29.70 (19.33–49.48) ng/mL vs. 5.83 (4.21–8.29) ng/mL, *p* < 0.001) (Figure 1).

**Figure 2:**
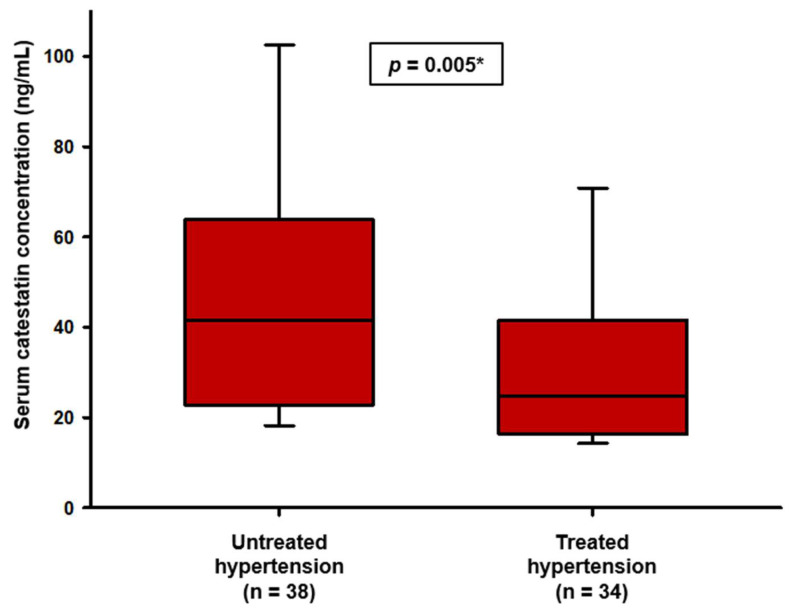
Comparison of catestatin concentrations between treated and untreated patients with primary hypertension. Data are presented as median and interquartile range. * Mann–Whitney U test.

The untreated subgroup of patients had significantly higher serum catestatin levels than patients treated with antihypertensive drugs (41.61 (22.85–63.83) ng/mL vs. 24.77 (16.41–40.21) ng/mL, *p* = 0.005) (Figure 2). Notably, the result remained significant even after adjustment to mean arterial pressure (F = 5.222, *p* = 0.008).

**Figure 3:**
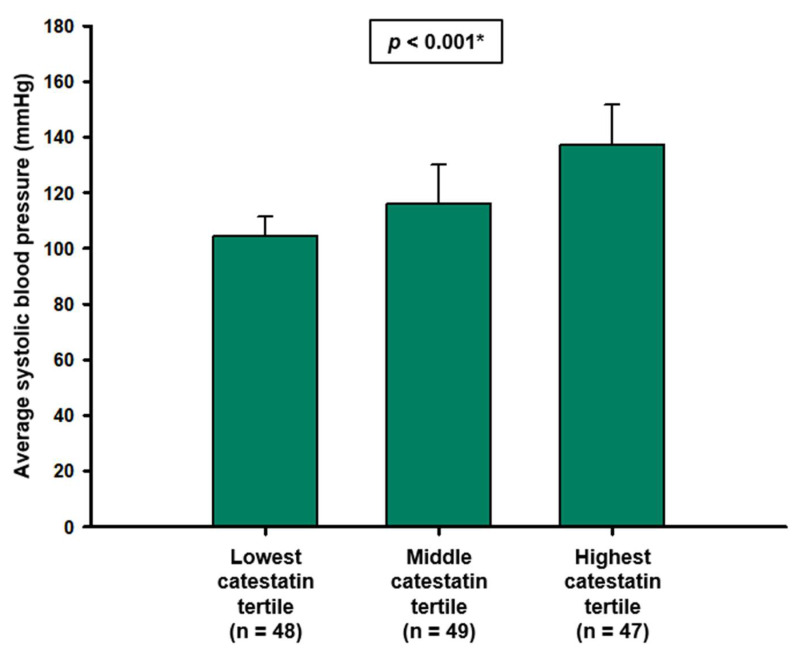
Comparison of ambulatory systolic blood pressures between groups based on catestatin terciles. * One-way ANOVA with *post hoc* Tukey test.

To explore an association between catestatin and ambulatory BP values, three distinct groups were created according to terciles of catestatin concentrations. The lowest levels of average SBP were observed in the tercile with the lowest catestatin levels, significantly higher in the second tercile, and the highest in tercile with the highest catestatin levels (104.4 ± 7.1 vs. 111.7 ± 10.7 vs. 128.1 ± 13.5 mmHg, *p* < 0.001) (Figure 3). Furthermore, a positive correlation was established between MAP and catestatin (r = 0.409, *p* < 0.001), and multiple linear regression analysis showed that serum catestatin levels retained significant association with MAP (β ± standard error, 0.8123 ± 0.3037, *p* = 0.009) after model adjustment for age and BMI.

**Figure 4:**
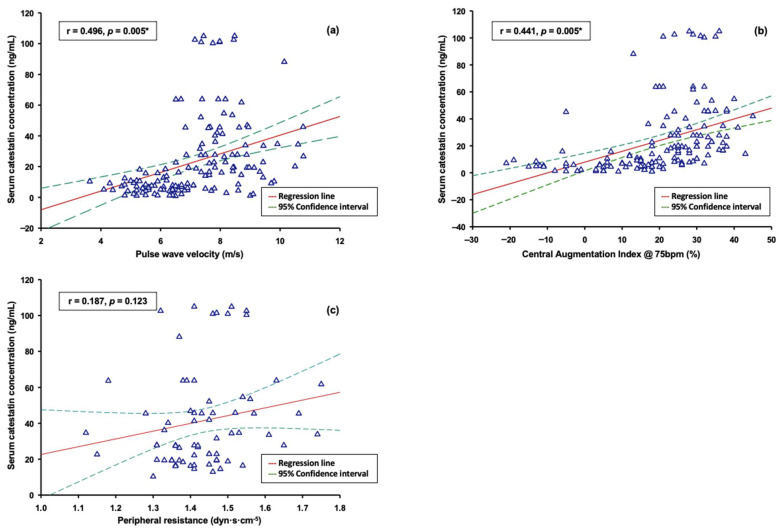
Correlation analysis between serum catestatin concentrations and indices of arterial stiffness: (**a**) correlation with PWV; (**b**) correlation with cAIx-75; (**c**) correlation with peripheral resistance. Abbreviations: PWV: pulse wave velocity; cAIx-75: central augmentation index at 75 beats-per-minute. * Spearman rank correlation analysis.

Finally, we explored an association between catestatin and the results of the PWA and AGEs. Serum catestatin levels positively correlated with PWV (r = 0.496, *p* < 0.001) and cAIx-75 (r = 0.441, *p* < 0.001), but not with pRes (r = 0.187, *p* = 0.123) (Figure 4a–c).

## 4. Discussion

In the present study, we established that serum catestatin concentrations in patients with mild to moderate PH are significantly higher in comparison to healthy controls matched by age, sex and BMI. Furthermore, in the subgroup of untreated patients with PH, catestatin levels were markedly higher than in the group of patients treated with ACE inhibitors and/or diuretics or in combination with CCBs and/or diuretics. Moreover, it was demonstrated that catestatin serum levels were higher in patients with higher ambulatory BP values, even after adjusting for age, sex and BMI. Finally, a moderate positive correlation was observed between indices of arterial stiffness and catestatin serum concentrations, but not with peripheral resistance. To the best of our knowledge, this is the first study in which the relation between ambulatory BP and catestatin serum concentrations was explored.

In a pivotal study, O’Connor et al. reported that serum catestatin levels in hypertensive patients do not differ from normotensive counterparts [19]. However, they also reported that catestatin levels were diminished in normotensive patients with a family history of hypertension (FH+) in comparison to FH- normotensive patients, despite comparable BP values between the groups. It is important to address that the hypertensive group from this study differed from the control group in multiple parameters that may affect serum catestatin. Most notable of these are age, BMI and blood glucose concentrations [20,21]. The authors did perform multiple linear regression to test whether the FH effect on catestatin was meaningful, but it was not excluded whether these differences may confound catestatin serum concentrations in patients with PH. In a subsequent study, the same authors demonstrated marginally higher levels of catestatin in the hypertension group, although it must be addressed that no adjustment to any of the now well-established factors that might influence catestatin levels was made [22]. In our study, we aimed to adjust for these confounders by establishing a control group matched by age, gender, BMI, fasting glucose, as well as HDL cholesterol, as correlation between HDL and catestatin has been previously established [23,24]. 

Nevertheless, the studies by O’Connor et al. address the complexity of catestatin implication in hypertension pathophysiology. Namely, it is presumed that in the early stages of hypertension, and even in prehypertension, low serum catestatin levels and the concomitant loss of its vasodilatory and catecholamine-inhibiting effects might contribute to the future development of PH. On the other hand, with the progression of the disease, catestatin levels may increase to compensatively inhibit the excessive secretion of catecholamines [25]. Schillaci et al. further expand these notions by claiming that catestatin may antagonize nicotinic cholinergic receptor desensitization, which occurs by prolonged exposure to its agonists, and thus even contributes to PH pathophysiology [26]. Accordingly, several studies showed that catestatin and Chromogranin A (ChgA) levels are significantly higher in hypertensive patients than in non-hypertensive patients in various populations [23,25,27,28]. Nevertheless, we found no correlation between catestatin and the duration of hypertension in our study, although it is also important to address that we measured time from diagnosis, and not time from disease onset. In addition, it is worth mentioning that Tüten et al. found higher catestatin levels in preeclampsia, an example of a relatively short-term hypertensive state [27].

Furthermore, in the present study, we found significantly higher catestatin serum levels in untreated patients when compared to healthy controls, even after adjustment for average MAP. According to the above-noted hypotheses, we would expect higher levels in treated patients, as disease duration was longer for these subjects. Two studies have so far reported a lack of difference in catestatin serum levels with respect to antihypertensive treatment [19,25]. Multiple factors could explain the discrepancy between our results and the available data. For start, in a study by Meng et al., patients were treated with CCBs and/or diuretics, whereas antihypertensive treatment in our study comprised mostly of ACE inhibitors, for which effects on SNS activity are established, unlike for the former two [29,30]. Additionally, it is worth mentioning that the vasoprotective effects of catestatin involve ACE2 [24]. On the other hand, O’Connor et al. had a relatively small untreated group (n = 13), and treatment regimes in the treated group were comprised of a palette of different antihypertensive medications, including beta-adrenergic antagonists, alpha1-adrenergic antagonists, alpha2-adrenergic agonists, angiotensin receptor antagonists and ACE inhibitors. Unfortunately, we did not include participants treated with CCBs or diuretics exclusively.

When we divided hypertensive patients in terciles according to catestatin levels, we observed that terciles with higher catestatin levels had significantly higher ambulatory BP values. Accordingly, a positive correlation between catestatin and ambulatory BP has been established and confirmed with multivariate analysis. These findings are in line with the available data and the aforementioned theories concerning the dynamic of catestatin with PH progression [27]. Yet, as previous studies have so far shown such association only with office BP values, and as it has been well-established that ambulatory BP is a more reliable indicator of BP, especially with regard to left ventricular hypertrophy (LVH), the present study substantially fortifies previous observations [31]. Nevertheless, the pathophysiological background behind this observation remains elusive. As previously noted, it is unclear whether catestatin serum levels rise with an increase in BP as a form of a “failed” compensatory mechanism or elevated catestatin, in fact, contributes to PH pathophysiology by sustaining catecholamine release. Specifically, preclinical studies imply that catestatin decreases BP in an acute setting by direct vasodilation and mediating central nicotinic acetylcholine receptors and β-adrenoceptors that are involved in cardiovascular regulation, whereas *ChgA*-gene knockout causes BP elevation that is normalized by intraperitoneal administration of catestatin in a rodent model [15,16,32,33,34]. On the other hand, catestatin was found to inhibit nicotine-induced desensitization of catecholamine release, thus sustaining the catecholamine release [35]. The authors of the aforementioned study hypothesize that this might be advantageous for the coping of humans with stress. Based on the established preclinical data, we may speculate that catestatin initially represents a compensatory mechanism aiming at BP reduction, which with time confers further progression of hypertension, especially in patients in which upregulated SNS activity underlies PH pathophysiology. Another point that requires attention is the eclectic nature of catestatin. Specifically, such high levels of catestatin that are observed in the present study are likely not a result of chromaffin cell secretion exclusively but rather reflect secretion from various tissues, such as the myocardium, further impeding the interpretation.

The results concerning PWA from the present study are in line with the available evidence. Specifically, in our previous study performed on the IBD population, we demonstrated that PWV, measured by applanation tonometry, positively correlated with serum catestatin levels, and that subgroup of patients with markers of end-organ damage (PWV > 10 m/s) had significantly higher levels of catestatin [36]. From a pathophysiological point of view, the association between PWV and catestatin is probably mediated by increased SNS activity, a mutual mechanism associated both with high catestatin levels and pathologic arterial remodeling, causing increased stiffness [37,38,39]. 

The present study bears several limitations. The study was designed as a single-center study and included only a Caucasian population, thus limiting the transfer of conclusion to other populations. As the study was cross-sectional, we may not discuss about causality. Nevertheless, for these reasons, we excluded patients with recent hypertensive crisis or other comorbidities which could bias our results, such as chronic diseases, diabetes, acute infections, smoking, etc. Finally, an additional group of patients treated with CCBs and/or diuretics would help in the delineation of effects attributable to ACE inhibitors with respect to serum catestatin concentrations. Perhaps the greatest strengths of the study are the facts that we used ambulatory, not office BP measurements, rendering the analysis more reliable, and that we carefully excluded patients with common comorbidities that accompany hypertension and might affect serum catestatin levels, such as obesity, metabolic syndrome and diabetes.

To summarize, in the present study, we established that serum catestatin concentrations are increased in hypertensive patients, especially in those treated for hypertension. Furthermore, for the first time, we described the positive correlation between ambulatory BP values and serum catestatin levels. Finally, a previously established correlation between catestatin and markers of arterial stiffness has now been demonstrated in patients with hypertension as well. Nevertheless, this being a single-center study and bearing in mind the high prevalence of PH, larger population-level studies are needed to establish whether catestatin bears diagnostic, therapeutic or prognostic value for patients with hypertension. In this regard, the pleiotropic nature of catestatin makes it even harder to explore, but given its potential, we find it important to gain further insight into the role of catestatin in cardiovascular disorders.

## Figures and Tables

**Table 1:** Baseline characteristics of the study population.

Characteristic	Control Group (n = 72)	Hypertension Group (n = 72)	*p*
Age, years	57.2 ± 9.2	54.6 ± 8.8	0.652 *
Male sex, n (%)	41 (56.9%)	45 (62.5%)	0.498 **
Body mass index, kg/m^2^	26.6 ± 2.8	27.2 ± 2.6	0.772 *
Waist-to-hip ratio	0.95 ± 0.31	0.98 ± 0.36	0.879 *
Dyslipidemia, n (%)	43 (59.7%)	48 (66.7%)	0.388 **
Family history of CV disease, n (%)	35 (48.6%)	30 (41.7%)	0.402 **
Time since diagnosis, years	N/A	4 (2–6)	N/A
Ambulatory blood pressure			
SBP, mmHg	121.7 ± 9.2	134.7 ± 13.0	<0.001 *
DBP, mmHg	78.4 ± 6.8	84.3 ± 10.3	<0.001 *
MAP, mmHg	91.5 ± 6.9	104.5 ± 10.8	<0.001 *
Pulse wave velocity, m/s	6.37 ± 1.43	8.05 ± 1.17	<0.001 *
End-organ damage ^1^, n (%)	2 (2.8%)	4 (5.6%)	0.681 ***
Fasting blood glucose, mmol/L	5.1 ± 0.7	5.2 ± 0.6	0.083 *
Cholesterol, mmol/L	5.24 ± 1.19	5.61 ± 0.97	0.045 *
LDL, mmol/L	3.27 ± 1.07	3.43 ± 0.86	0.315 *
HDL, mmol/L	1.41 ± 0.32	1.51 ± 0.45	0.151 *
Triglycerides, mmol/L	1.22 ± 0.63	1.54 ± 0.90	0.014 *

Data presented as mean ± standard deviation or n (%) ^1^ According to the ESC/EHA guidelines, pulse wave velocity > 10 m/s is considered to represent end-organ damage. * Student’s test ** Chi squared test *** Fisher’s exact test Abbreviations: SBP: systolic blood pressure; DBP: diastolic blood pressure; MAP: mean arterial pressure; LDL: low-density lipoprotein; HDL: high-density lipoprotein; CV: cardiovascular.

**Table 2:** Ambulatory blood pressure and pulse wave analysis comparison between the treated and untreated group.

Parameter	Untreated Group (n = 38)	Treated Group (n = 34)	*p*
Average SBP, mmHg	135.4 ± 13.5	133.9 ± 12.6	0.632 *
Average DBP, mmHg	85.9 ± 11.0	82.3 ± 9.0	0.144 *
Average MAP, mmHg	105.8 ± 11.5	102.9 ± 9.9	0.265 *
Awake SBP, mmHg	140.6 ± 13.1	137.8 ± 12.3	0.368 *
Awake DBP, mmHg	90.4 ± 10.9	85.9 ± 8.7	0.264 *
Awake MAP, mmHg	110.6 ± 11.0	106.6 ± 9.5	0.113 *
Nocturnal SBP, mmHg	126.9 ± 17.2	127.5 ± 15.4	0.884 *
Nocturnal DBP, mmHg	78.5 ± 13.9	76.3 ± 11.1	0.482 *
Nocturnal MAP, mmHg	97.9 ± 14.7	96.9 ± 12.3	0.772 *
Dipper, n (%)	22 (58%)	16 (47%)	0.361 **
Time since diagnosis, years	3 (2–4)	5 (4.3–7.8)	<0.001 ***
Hypertension stage			
Mild, n (%)	29 (76.3%)	30 (88.2%)	0.192 **
Moderate, n (%)	9 (23.7%)	4 (11.8%)	
Pulse wave velocity, m/s	7.98 ± 1.22	8.13 ± 1.13	0.604 *
Peripheral resistance, dyn·s·cm^−5^	1.44 ± 0.11	1.42 ± 0.13	0.488 *
cAIx@75bpm, %	30.1 ± 6.3	28.7 ± 7.9	0.424 *

Abbreviations: SBP: systolic blood pressure; DBP: diastolic blood pressure; MAP: mean arterial pressure; cAIx@75bpm: central augmentation index at 75 beats-per-minute. * Student’s *t*-test ** χ^2^ test *** Mann–Whitney U test.

## Data Availability

The data presented in this study are available on request from the corresponding author. The data are not publicly available because some of the data set will be used for further research.
